# Total Ionizing Dose Effect Simulation Study on 130 nm CMOS Processor

**DOI:** 10.3390/mi17010132

**Published:** 2026-01-20

**Authors:** Yi Liu, Yuchen Liu, Xinfang Liao, Changqing Xu, Yangchen He, Yintang Yang

**Affiliations:** 1 Shenzhen Institute of Technology, Xidian University, Shenzhen 518000, China; yiliu@mail.xidian.edu.cn; 2 Faculty of Integrated Circuit, Xidian University, Xi’an 710071, China; 23211215184@stu.xidian.edu.cn (Y.L.); ytyang@xidian.edu.cn (Y.Y.); 3 Guangzhou Institute of Technology, Xidian University, Guangzhou 510555, China; cqxu@xidian.edu.cn (C.X.); hyc198519218072022@163.com (Y.H.)

**Keywords:** total ionizing dose effect, 130 nm bulk CMOS, processors, system-level simulation, failure threshold

## Abstract

This paper reports the results of a system-level total ionizing dose (TID) effect simulation study on a SMIC 130 nm LEON2 processor. Firstly, the device-level simulations of the 130 nm NMOS transistors are performed using the Sentaurus TCAD software to analyze the effects of a bias condition, channel width, and irradiation dose on a TID-induced leakage current. Based on the TCAD simulation results, a Verilog-A-based compact model is developed for NMOS transistors to describe the TID-induced leakage current, and it is then embedded into target nodes of the SPICE netlist for the LEON2 processor, enabling system-level TID simulations. The simulation results reveal the processor’s failure threshold and corresponding failure mechanism; meanwhile, the increase in the power supply current with the irradiation dose is also observed. The research reported in this paper can provide beneficial guidance for radiation performance evaluation and radiation hardening by design (RHBD) in 130 nm bulk CMOS processors.

## 1. Introduction

With the rapid development of the aerospace industry, the complexity of the spacecraft mission and in-orbit operation time has increased significantly [1]. And, the total ionizing dose (TID) effect, as a cumulative effect, is widely present in a low-earth orbit, medium-earth orbit, geosynchronous transfer orbit, and deep space exploration environment, and has become a key factor restricting the life of aerospace devices and circuits [2]. Beyond space, the high-radiation environments characteristic of High-Energy Physics (HEP) facilities, such as particle accelerators and detector areas, also present extreme TID challenges, making radiation hardness a fundamental requirement for electronics operating in such settings [3].

As an important research methodology, total ionizing dose effect simulation plays a key role in revealing the failure mechanism and guiding radiation-hardened design. At present, researchers have extensively investigated TID modeling and simulation methods [4,5,6]. Esqueda et al. completed the modeling of the oxide trap charge and the interface trap charge by calculating the surface potential at each position of the channel, which can better describe the working characteristics of an MOS device under a TID effect [7]. Gao T et al. established a model of a CMOS inverter circuit based on the Sentaurus TCAD software [8], and used a mixed-mode simulation method to add the radiation model to the circuit, so as to study and analyze the influence of the TID effect on the CMOS inverter circuit [9].

However, existing total ionizing dose effect simulation studies predominantly focus on the individual transistors and small-scale circuits, with there being limited reports on processor-class large-scale digital ICs [9]. As the core module of space-borne computer systems, rapid and effective assessment of a processor’s radiation hardness is critical. Although experimental methods can accurately evaluate its performance in specific radiation environments, these are time-consuming and costly, while failing to reveal the failure mechanisms and guide the subsequent radiation hardening by design (RHBD) [10,11].

To address this technique challenge, this paper reports on a simulation study on the TID effect of the LEON2 processor (European Space Agency (ESA), Paris, France), which is a typical processor module in space-borne computers. The LEON2 processor is a processor designed for aerospace embedded applications. It adopts the SPARC V8 RISC architecture and offers advantages such as operational stability, strong scalability, and an open architecture. It has been widely used in the aerospace sectors of Europe and the United States [12,13]. We first employ the TCAD simulation software to investigate the TID-induced leakage current as a function of the bias condition, channel width, and irradiation dose for an NMOS transistor and on this basis, we conduct full-system TID effect simulation for the SMIC 130 nm LEON2 processor (Semiconductor Manufacturing International Corporation (SMIC), Shanghai, China). Our research reveals the functional failure threshold and allows for observation of the increase in power supply current with irradiation dose. Meanwhile, through simulations of standard cell circuits, the failure mechanism is systematically analyzed.

## 2. TCAD Simulation and Fault Injection Model Establishment

A pronounced difference in TID susceptibility exists between NMOS and PMOS transistors, rooted in their opposing mechanistic behaviors. The buildup of positive trapped charges in the oxide has contrasting consequences: for NMOS transistors, it results in a negative threshold voltage shift, severely elevating off-state leakage [14,15]. In contrast, for PMOS transistors, this positive charge buildup drives the threshold voltage to being even more negative. This shift beneficially reduces the PMOS leakage current and lessens the potential for parasitic conduction in CMOS logic circuits [16,17]. Additionally, the generally lower operational gate bias in PMOS devices diminishes the oxide electric field, thereby causing much less net charge trapping compared to their NMOS counterparts [18]. Thus, at the total dose levels of interest, PMOS transistors do not suffer significant electrical degradation. Since the TID-induced degradation of circuits is predominantly governed by the NMOS characteristics, the scope of this study is confined to the analysis and modeling of TID effects in NMOS transistors.

**Figure 1:**
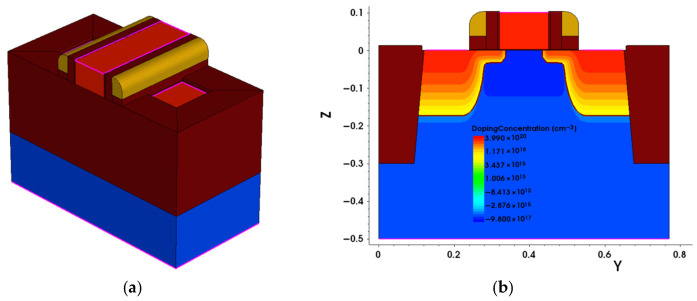
3D TCAD model for NMOS transistor. (**a**) A 3D view of transistor. (**b**) Cross-sectional view at the channel, W = 150 nm, L = 130 nm.

**Figure 2:**
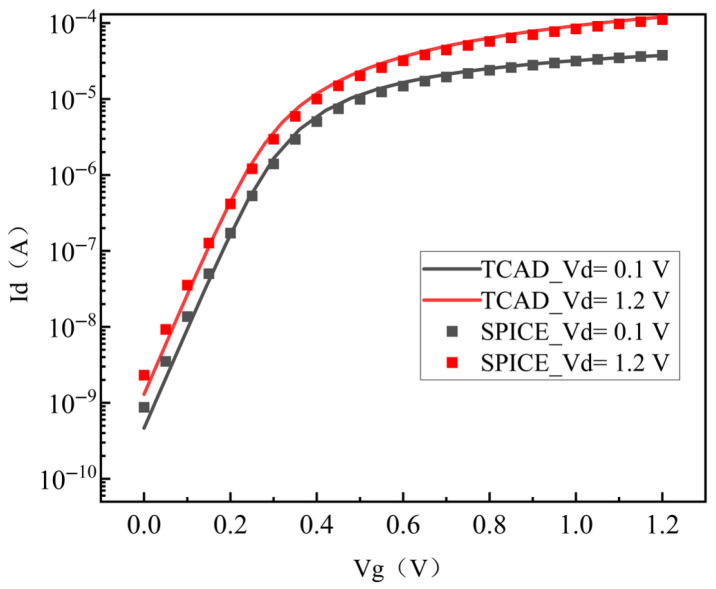
Comparison of transfer characteristics between the TCAD model and SPICE model.

Based on the SMIC 130 nm device structure and parameters, a three-dimensional model of an NMOS transistor is established by Sentaurus TCAD software 2018.06-SP2, as shown in Figure 1 (W/L = 150 nm/130 nm). To accurately simulate the actual degradation condition, important device parameters such as the doping concentration and diffusion coefficient are calibrated. Eventually, the optimized TCAD device model demonstrates excellent agreement with the SPICE model in terms of the transfer characteristics, which is illustrated in Figure 2.

As the ionizing irradiation with high energy acts on the MOS transistor, electron-hole pairs are generated in the oxide, which induces the accumulation of trapped charge and is responsible for device performance degradation [19]. The radiation type in this study is X-ray, with a total dose of 1 Mrad. To obtain accurate and physically consistent research results, we performed TID simulations on NMOS transistors by implanting a fixed positive charge at the Si/SiO_2_ interface through the Insulator Fixed-Charge model [20], with parameters calibrated based on the experimental leakage current data of NMOS transistors (W/L = 150 nm/130 nm) in 130 nm technology [18,21].

**Figure 3:**
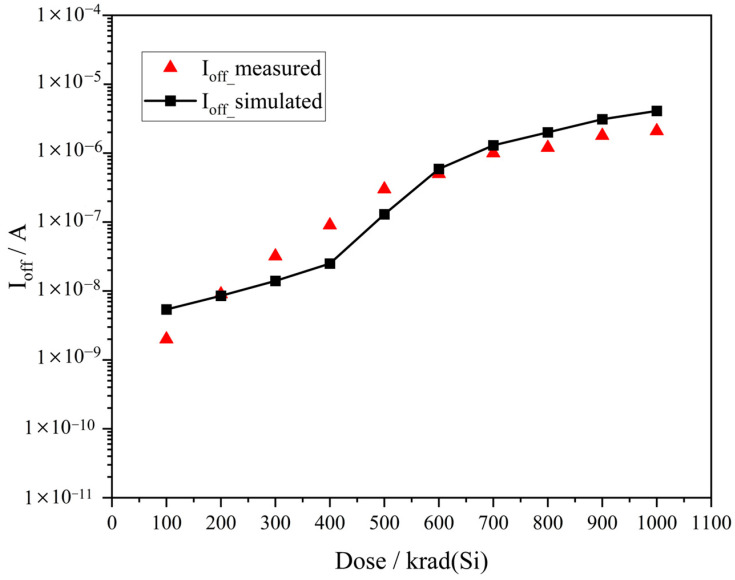
Comparison between TCAD-simulated and experimentally measured off-state leakage current data.

Figure 3 compares the simulated off-state leakage current data under different irradiation doses (obtained by implanting varying concentrations of a fixed positive charge in the 3D model) with the experimental data [18,21]. As we can see, the simulation results are consistent with the experimental data, indicating that the established TCAD simulation model can effectively simulate the TID effect.

**Figure 4:**
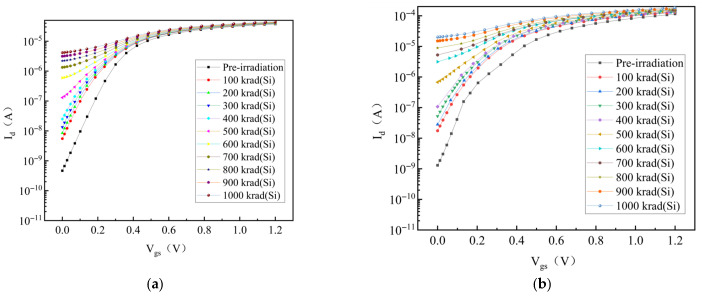
TID-induced leakage current simulation results for NMOS transistor (W/L = 150 nm/130 nm). (**a**) V_d_ = 0.1 V; (**b**) V_d_ = 1.2 V.

When the processor is operating, the NMOS transistors will be in different bias states, so the influence of the bias state on the TID-induced leakage current should also be considered. The bias conditions for the transfer characteristics are V_d_ = 0.1 V and 1.2 V, with V_gs_ swept from 0 V to 1.2 V, and the simulation results are shown in Figure 3. We can observe from Figure 4 that the device exhibits varying degrees of degradation with the irradiation dose, especially in the subthreshold region. Moreover, the increase in leakage current after radiation under high-drain voltage is significantly higher than that under low-drain voltage, because the high-drain voltage significantly enhances the conduction capacity of the parasitic channel. As a result, the influencing factor of V_d_ must be taken into account when modeling the TID-induced leakage current in an NMOS transistor.

**Figure 5:**
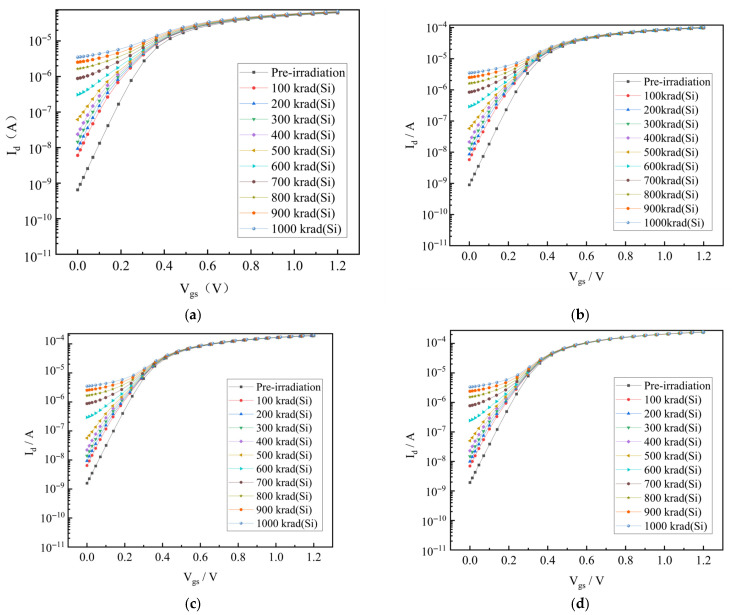
TID simulation results for NMOS transistors with different channel widths. (**a**) W = 250 nm; (**b**) W = 400 nm; (**c**) W = 800 nm; (**d**) W = 1000 nm.

NMOS transistors in the processor core maintain an identical channel length (L = 130 nm) while implementing varied channel widths to meet drive current requirements. Based on this, we also modelled the TID-induced leakage current as a function of the channel width. Figure 5 shows the transfer characteristic curves of devices with channel widths of 250 nm, 400 nm, 800 nm, and 1000 nm under different irradiation doses. The simulation results show that the leakage current increment of devices with different channel widths is almost the same under the same irradiation dose when L is fixed. This phenomenon is consistent with the experimental results reported in the literature [22,23]. Therefore, in the subsequent research, we will ignore the effect of the channel width on the TID-induced leakage current, and equate the TID-induced leakage current in NMOS transistors with different aspect ratios to that in the NMOS transistor with W/L of 150 nm/130 nm.

Based on the above work, we introduced the parasitic transistor to model the TID-induced leakage current [24]. During parasitic current calculation, the SPICE Level 3 MOS model is modified, taking into account the actual structural disparities as well as process parameter uncertainties. Through iterative optimization of model parameters, we introduce radiation-dependent fitting coefficients ( $k_{1}$, $k_{2}$) into both the saturation current equation and saturation voltage expression, effectively correlating leakage current data under varying irradiation doses and bias conditions. Finally, the modified formulations are as follows:

(1) $$Y_{E}={\left(\frac{\left|E\right|+E_{0}}{\left|E\right|+E_{1}}\right)}^{m}$$

where $Y_{E}$ is the hole yield, E is the electric field intensity, $E_{0}$ = 0.1 V/cm, $E_{1}$ = 1.35 MV/cm, and m = 0.9.

(2) $$t_{ox}\left(z\right)=\frac{z}{\sin\left(\theta \right)}\cdot 2\pi \cdot \frac{\theta }{360}$$

where $t_{ox}$ is the equivalent gate oxide thickness, and θ and z are the sidewall angle and depth of the STI trench, respectively.

The trap charge density can be expressed as follows:

(3) $$p_{T}\left(z\right)=N_{T}\left[1-exp\left(-\sigma_{P}\cdot g_{0}\cdot D\cdot Y_{E}\cdot t_{ox}\left(z\right)\right)\right]$$

where $N_{T}$ is the concentration of the neutral trap in oxide layer (≈ 3 × 10^17^ cm^−3^), $\sigma_{P}$ is the cross-section of the hole trapped by the oxide neutral trap (≈ 5 × 10^−12^ cm^2^), D is the total dose, and $g_{0}$ is the electron-hole pair generated by radiation in the oxide (≈ 7.8 × 10^12^ rad^−1^ cm^−3^).

The threshold voltage of the parasitic transistor at depth z can be modeled pre- and post-irradiation as follows:

(4) $$V_{T0}\left(z\right)=\phi_{S^{\prime }S}-\frac{-\sqrt{2q\varepsilon_{Si}N_{A}\left(z\right)\cdot 2\psi_{F}\left(z\right)}}{\varepsilon_{SiO_{2}}/t_{ox}\left(z\right)}+2\psi_{F}\left(z\right)$$

(5) $$V_{T}\left(z\right)=V_{T0}\left(z\right)-\frac{p_{T}\left(z\right)\cdot q\cdot d}{\varepsilon_{SiO_{2}}/t_{ox}\left(z\right)}$$

where $\phi_{S^{\prime }S}$ is the difference in work function between the gate electrode and substrate, $\psi_{F}\left(z\right)$ is the Fermi potential, and d is the equivalent length when the trap charge obeys a uniform distribution.

(6) $$W=\frac{\Delta z}{\sin\left(\theta \right)}$$

(7) $$I_{D}=0,V_{GS}<V_{T}\left(z\right)$$

(8) $$I_{D}\left(z\right)=\frac{W\frac{\varepsilon_{SiO_{2}}}{t_{ox}\left(z\right)}\left(V_{GS}-V_{T}\left(z\right)-\frac{V_{DS}}{2}\right)V_{DS}}{L\left(1+\frac{\mu_{lf}V_{DS}}{Lv_{sat}}\right)},V_{DS}\leq V_{Dsat},V_{GS}\geq V_{T}\left(z\right)$$

(9) $$I_{D}\left(z\right)=k_{2}W\frac{\varepsilon_{SiO_{2}}}{t_{ox}\left(z\right)}v_{sat}\left(V_{GS}-V_{T}\left(z\right)-V_{Dsat}\right)V_{DS},V_{DS}>V_{Dsat},V_{GS}\geq V_{T}\left(z\right)$$

(10) $$V_{Dsat}=k_{1}\frac{v_{sat}}{\mu_{lf}}L\left[{\left(1+\frac{2\mu_{lf}\left(V_{GS}-V_{T}\left(z\right)\right)}{v_{sat}L}\right)}^{1/2}-1\right]$$

(11) $$\mu_{lf}=\frac{\mu_{n}}{1+\theta \left(V_{GS}-V_{T}\left(z\right)\right)}$$

(12) $$I_{p}=\int_{0}^{z_{max}}I_{D}\left(z\right)dz$$

where $\mu_{lf}$ is the low field mobility and $v_{sat}$ is the carrier saturation velocity.

**Figure 6:**
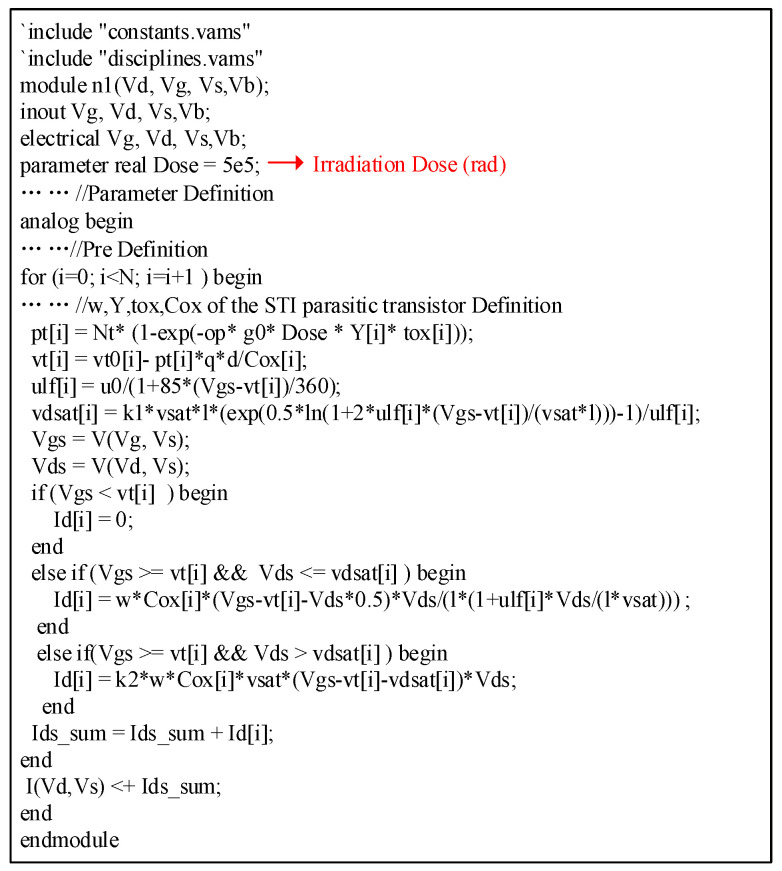
TID-induced leakage current model implemented in Verilog-A.

**Figure 7:**
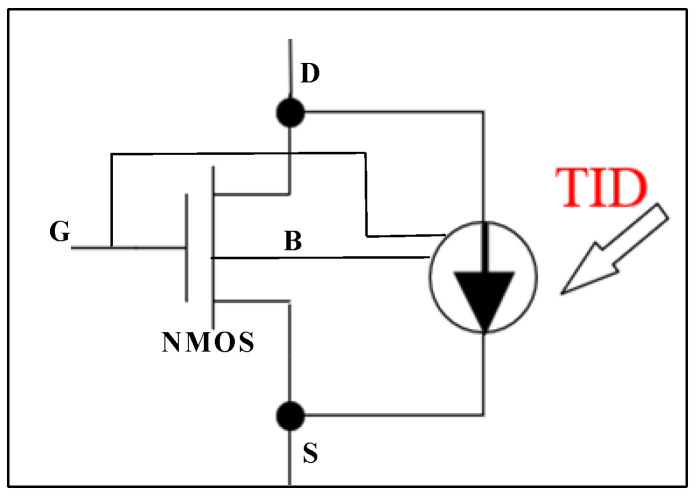
Schematic implementation of circuit-level fault injection model.

**Figure 8:**
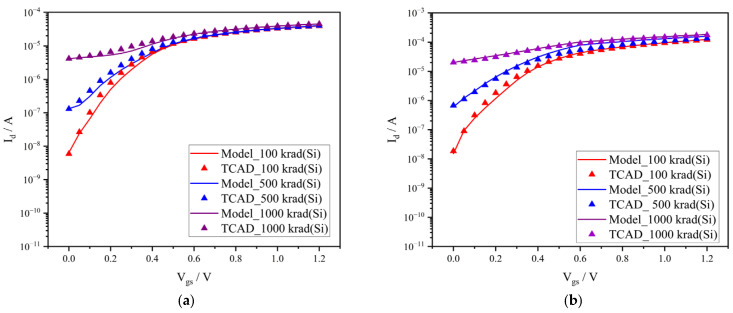
Comparison in TID-induced leakage current between TCAD simulations and SPICE circuit simulations. (**a**) Vd = 0.1 V; (**b**) Vd = 1.2 V.

We use the Verilog-A programming language to embed the parasitic current model into the standard simulation tool SPICE, as shown in Figure 6, and attach the parasite current to the main current of the NMOS transistor, as shown in Figure 7. Therefore, we can obtain the electrical characteristics of the NMOS transistor after irradiation in circuit-level simulations. Figure 8 shows the comparison in TID-induced leakage current between the TCAD simulation results and the SPICE simulation results, from which it can be seen that the Verilog-A-based model can accurately describe the degradation in NMOS transistors at different irradiation doses and biases.

## 3. Circuit-Level Simulation Results and Discussion

After the synthesis of the LEON2 processor’s RTL code and its conversion to a post-layout SPICE netlist, the physical implementation typically comprises approximately 40,000 cells and occupies a silicon area of about 560,000 μm^2^. The developed fault injection model is then embedded into all MOS transistor target nodes in the resulting netlist, enabling the simulation of TID-induced performance degradation of the processor at the circuit level.

The Fibonacci sequence calculation is chosen as the test benchmark. This program is selected for its clear computational steps, comprehensive coverage of data paths, and easily verifiable results, making it effective for examining the functional correctness of the processor under the TID radiation effect. The criterion for functional failure is defined as the inability to execute the program or to correctly write the final result to the data SRAM. The expected correct result for the program is the Fibonacci sequence: 1, 2, 3, 5, 8, 13, 21, 34 [25].

**Figure 9:**
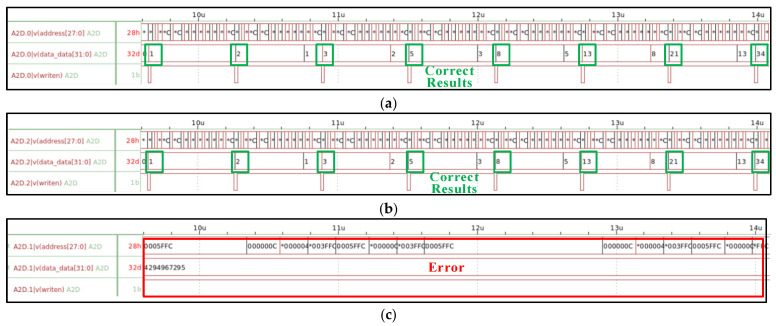
System simulation waveforms at different irradiation doses: (**a**) pre-irradiation, (**b**) 760 krad(Si), and (**c**) 770 krad(Si). Due to the limited display resolution during the zooming process of the simulation waveform, some non-critical numerical details could not be fully rendered. Therefore, the asterisk symbol (*) is used in the figure to indicate that the numerical values at those positions have been truncated due to insufficient display precision. The key nodal waveform values are clearly presented, and this display issue does not affect the qualitative analysis conclusions of the simulation results in this paper.

The simulation results of the processor under different irradiation doses are shown in Figure 9. As we can see, the processor maintains correct functional operation and accurate computational outputs as the irradiation dose increases from 0 krad(Si) to 760 krad(Si). However, when the irradiation dose reaches 770 krad(Si), the processor experiences an irrecoverable functional failure. Specifically, the processor’s write signal (writen) and the data SRAM’s output signals (data_data [31:0]) remain stuck in their initial states. Concurrently, the address bus (address [27:0]) exhibits unexpected toggling or becomes locked. These observations indicate that the processor has lost its ability to control the memory correctly, rendering it incapable of performing valid read or write operations. Consequently, the system functionality completely breaks down.

**Figure 10:**
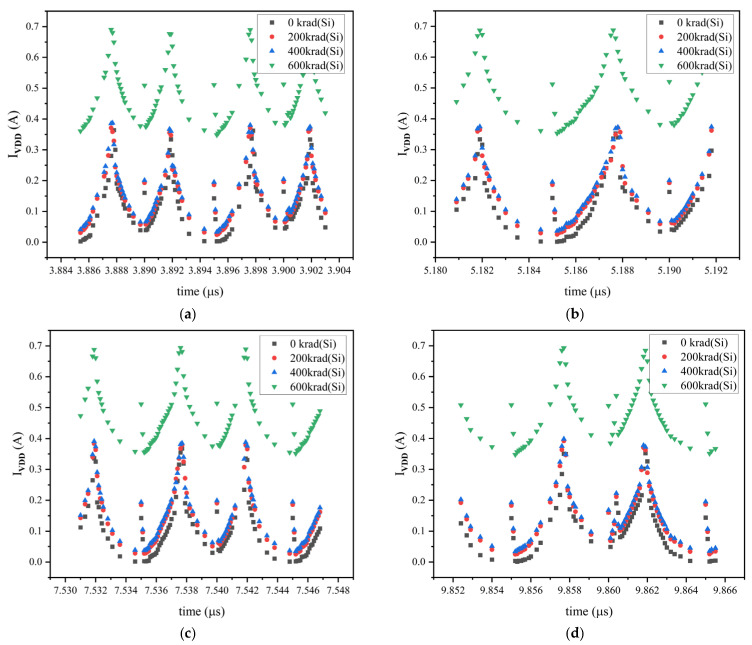
Variations in power supply current over several specific time periods under different irradiation doses. (**a**) 3.884 μs to 3.904 μs; (**b**) 5.180 μs to 5.192 μs; (**c**) 7.530 μs to 7.548 μs; (**d**) 9.852 μs to 9.866 μs.

The impact of the TID effect on power consumption is a key metric for assessing the LEON2 processor’s radiation reliability, as its variation directly reflects the degradation of physical characteristics of the internal transistors in the processor [26,27,28]. To characterize the increase in power consumption of the processor under the radiation environment, we monitor the system’s power supply current during the simulation process. Figure 10 shows the variations in the power supply current of the LEON2 processor system over several specific time periods under different irradiation doses. From the simulation results, we can observe that as the irradiation dose increases from 0 krad(Si) to 600 krad(Si), the system’s power supply current exhibits a significant increase. At irradiation doses of 200 krad(Si) and 400 krad(Si), the amplitude of the power supply current changes slightly, and the processor system will not experience functional failure. However, when the irradiation dose increases to 600 krad(Si), the power supply current fluctuates violently. It can be seen from Figure 10 that the power supply current increases even beyond 100% at 600 krad(Si), strongly indicating severe degradation in the switching characteristics of internal circuits, such as logic gates and flip-flops.

To quantify the impact of the TID effect on the LEON2 processor’s power consumption, we systematically analyze the power supply current based on transient simulation results at different irradiation dose levels. Using a dedicated data processing script, the total operating current of the processor under each irradiation dose is automatically extracted, and the corresponding average power consumption is calculated. This process yields the quantitative relationship between the average power supply current and the increasing irradiation dose, as Figure 10 shows.

**Figure 11:**
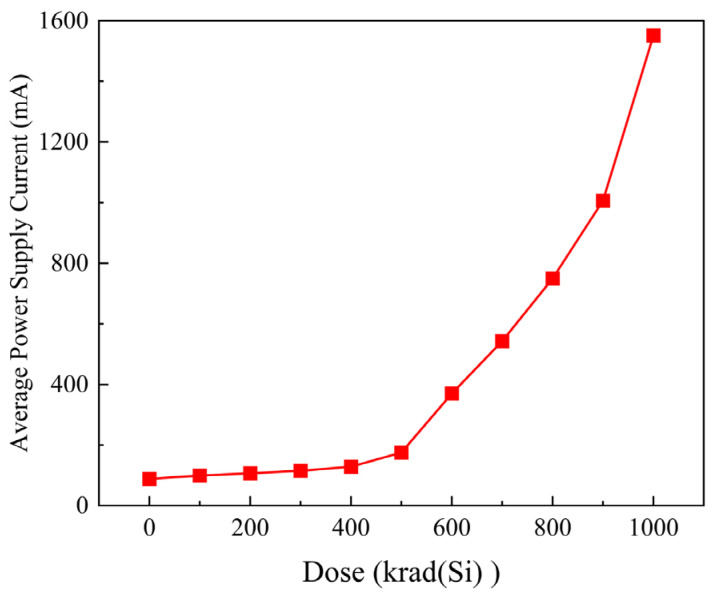
Average power supply current of the LEON2 processor system versus irradiation dose.

A significant positive correlation between the power supply current and the irradiation dose is clearly observable from Figure 11: the current exhibits a continuous rise as the irradiation dose accumulates. Specifically, within the irradiation dose range of 0 krad(Si) to 400 krad(Si), the power supply current increases in an approximately linear trend. This indicates a relatively moderate level of the radiation-induced damage during this phase, with a steady increase in leakage current. However, when the total irradiation dose reaches the interval of 400 krad(Si) to 500 krad(Si), the growth rate of the power supply current shows a marked increment. This indicates severe degradation in the system performance, signifying that the radiation-induced damage has transitioned into an accelerated phase. With a further increase in irradiation dose, the current’s growth rate continues to intensify, demonstrating a characteristic nonlinear degradation pattern. The trend of the increasing power supply current with the irradiation dose, as revealed by the simulation, is in excellent agreement with the experimental data, which exhibits a typical three-stage variation regulation of “steady linear increase—accelerated degradation—dramatic nonlinear degradation” [29,30,31].

From the fundamental mechanism perspective, the TID effect induces transistor-level performance degradation, such as a decrease in the threshold voltage and an increase in the leakage current. At the cell circuit level, this degradation manifests as shifts in logic levels and the deterioration of timing parameters. Since the system-level circuit is composed of a large number of basic standard cell circuits, the performance degradation at the standard cell circuits is amplified stage by stage at the system level, ultimately leading to functional failure of the entire processor [32]. Hence, to systematically analyze the root cause leading to the processor’s failure under the TID effect, it is necessary to conduct simulation modeling and a characteristic analysis of cell circuits. In this study, TID simulation research is performed for all standard cell circuits used in the LEON2 processor, including combinatorial logic cells and sequential logic cells. Table 1 and Table 2 summarize their failure thresholds.

**Figure 12:**
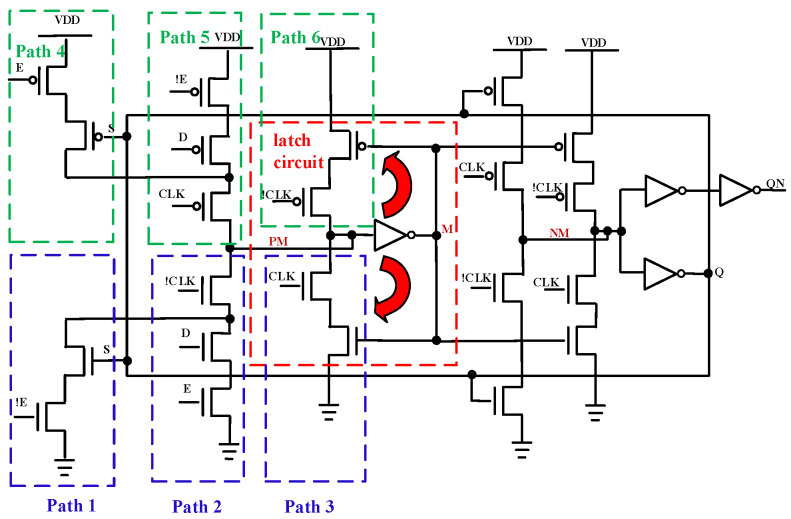
Schematic diagram of the EDFFX1TL-register circuit structure.

**Figure 13:**
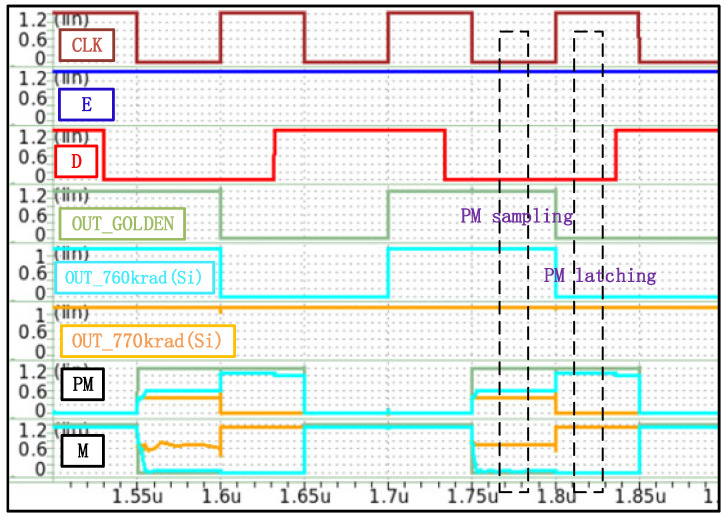
Simulation waveforms of EDFFX1TL register under different irradiation doses.

According to the data in Table 1 and Table 2, the EDFFX1TL register exhibits the lowest failure threshold among all standard cell circuits, with a value of 770 krad(Si). This value is fully consistent with the overall system failure threshold of the processor. To investigate the EDFFX1TL register’s detailed damage mechanism, Figure 12 and Figure 13 show its circuit structure and simulated waveforms under different irradiation doses, respectively. It can be seen from Figure 12 that in the PM node (an internal storage node of the flip-flop) sampling stage, due to the influence of the TID effect, there are three pull-down leakage paths. Therefore, the PM node voltage will gradually decrease with the cumulative irradiation dose. When the cumulative dose reaches 760 krad(Si), as Figure 13 shows, the PM node voltage decreases to approximately VDD/2. But, at this time, the M node voltage can still be repaired to an ideal low level by the inverter. As a result, when the CLK signal jumps to the high level, the self-locking path can still maintain its normal function. In contrast, when the cumulative irradiation dose is increased to 770 krad(Si), the voltage of the PM node further decreases, and the inverter connected with the PM node has no ability to repair, so the output voltage of the M node exhibits a significant increase, even exceeding VDD/2. At the rising edge of the CLK signal, the pull-down path 3 is gradually turned on, and the leakage current exhibits more significant degradation. At this time, the self-locking path will form a feedback mechanism, so that the PM node will completely lose its latch function and output a low level, resulting in the register being unable to correctly store the input data and the function of the register completely failing.

In the LEON2 processor architecture, registers are the core components for instruction execution and data processing, as they store crucial signals such as instructions, data, and control information. The EDFFX1TL register is the most frequently used register in the LEON2 SPICE netlist, which runs throughout the data and control paths of the processor. When the irradiation dose reaches 770 krad(Si), the EDFFX1TL register fails and the program counter (PC) is no longer able to correctly store the address of the next instruction to be executed, so there will be an error in the address data, which will cause the system to crash. Therefore, based on the simulation waveforms and the above analysis, we attribute the severe functional failure of the processor under the irradiation dose of 770 krad(Si) to the incorrect data store and transfer in the EDFFX1TL register.

**Figure 14:**
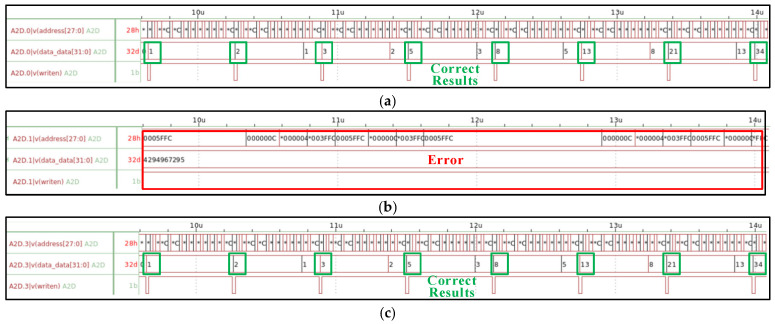
System simulation waveforms at different irradiation doses: (**a**) pre-irradiation, (**b**) 770 krad(Si), and (**c**) 770 krad(Si) (without fault injection in the EDFFX1TL register). Due to the limited display resolution during the zooming process of the simulation waveform, some non-critical numerical details could not be fully rendered. Therefore, the asterisk symbol (*) is used in the figure to indicate that the numerical values at those positions have been truncated due to insufficient display precision. The key nodal waveform values are clearly presented, and this display issue does not affect the qualitative analysis conclusions of the simulation results in this paper.

To verify the above inference, a comparative simulation scheme is designed and presented in this paper: except for NMOS transistors in the EDFFX1TL register, all NMOS transistors in the LEON2 processor are injected with the circuit-level fault model, and the simulation results at the irradiation dose of 770 krad(Si) are illustrated in Figure 14. It can be clearly observed that when no fault model is injected into the EDFFX1TL register, the processor system can operate normally without any error, as Figure 14c shows. Whereas, as mentioned above, when the fault injection model is applied to all cell circuits in the LEON2 processor at the same irradiation dose, the processor exhibits obvious functional failure, as Figure 14b shows. The direct comparison between these two sets of simulation results confirms that the EDFFX1TL register is the root cause leading to the processor’s system-level functional failure at the irradiation dose of 770 krad(Si).

**Figure 15:**
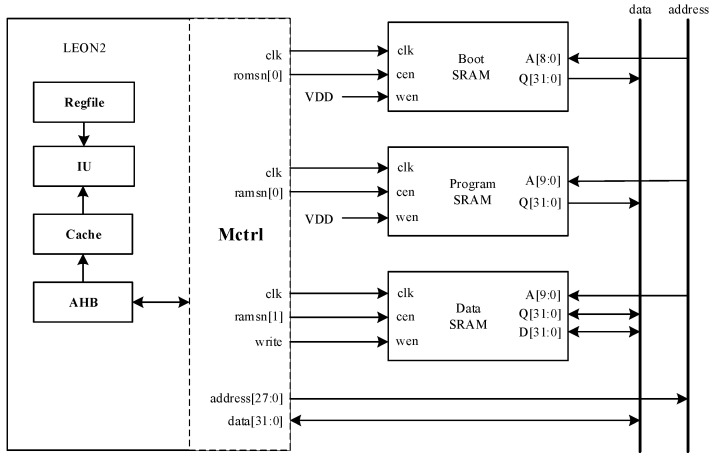
Schematic diagram of internal fault propagation in the LEON2 processor architecture.

Based on the LEON2 processor architecture illustrated in Figure 15, the system-level fault propagation mechanism can be further elucidated. As is shown in Figure 15, the register file (Regfile), serving as the core storage unit of the processor, integrates a significant number of EDFFX1TL register structures. When the total irradiation dose reaches 770 krad(Si), the EDFFX1TL register fails first and becomes the initial source of the system-level fault, due to its relatively low radiation tolerance. Following the EDFFX1TL register’s failure, the erroneous signal propagates rapidly through the internal data path of the register file to the memory controller (Mctrl). Since the Mctrl module is responsible for coordinating the processor’s access to various memory units (including the boot SRAM, program SRAM, and data SRAM), anomalies in its input signals directly lead to errors in address generation and read/write control logic. The fault subsequently propagates through the AHB and cache subsystem to the memory hierarchy, causing errors in instruction fetch and failures in data access, ultimately resulting in the complete functional collapse of the processor.

**Figure 16:**
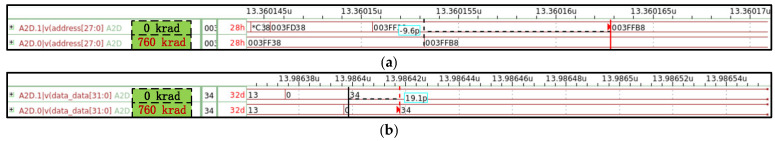
A comparison of timing fluctuations in critical port signals at 760 krad(Si) with the pre-injection baseline waveform: (**a**) address [27:0], (**b**) data_data [31:0]. Due to the limited display resolution during the zooming process of the simulation waveform, some non-critical numerical details could not be fully rendered. Therefore, the asterisk symbol (*) is used in the figure to indicate that the numerical values at those positions have been truncated due to insufficient display precision. The key nodal waveform values are clearly presented, and this display issue does not affect the qualitative analysis conclusions of the simulation results in this paper.

In complex radiation environments, the timing stability of a processor is crucial for ensuring system reliability. To evaluate its timing characteristics, we conduct detailed observations of the LEON2 processor at a radiation dose of 760 krad(Si). As is shown in Figure 16, timing drift occurs in both the processor’s address signals (address [27:0]) and SRAM data output signals (data_data [31:0]). Precise measurements using waveform cursors reveal drift values of –9.6 ps and +19.1 ps, both at the picosecond level. Previous studies establish the functional failure dose threshold for this processor as being 770 krad(Si). Since the system remains fully functional at 760 krad(Si), the observed picosecond-level timing drift near the failure threshold is not the primary cause of functional failure.

To further evaluate the overall radiation tolerance of the processor under complex dynamic workloads, this study employed a multi-program simulation test, covering typical tasks such as integer operations, matrix multiplication, and bubble sorting, in order to overcome the limitations of single-scenario testing. The results show that the radiation dose threshold for processor functional failure is consistently 770 krad(Si), which is in agreement with the aforementioned findings. This further confirms the reliability of the simulation method and the validity of the threshold conclusion.

Based on the simulation results and analysis, a clear correlation is observed between the overall radiation tolerance of the processor and the radiation performance of its internal logic units. In line with this finding, during the RTL synthesis stage, standard cells with higher radiation resistance are prioritized for implementation based on their radiation simulation data. Moreover, localized hardening strategies—such as the adoption of radiation-hardened fabrication processes or annular gate transistor layouts—can be applied to the circuit modules that are most sensitive to the TID effect. This methodology ensures robust radiation protection for the processor while effectively controlling the area and power overhead that is introduced by the hardening measures, thereby achieving an optimal balance among performance, reliability, and cost.

## 4. Conclusions

A simulation study of the TID effect was carried out on the SMIC 130 nm LEON2 processor. The results show that the processor experiences functional failure under the irradiation dose of 770 krad(Si), accompanied by a sharp increase in the average power supply current beyond a specific dose threshold. Through the comprehensive simulation analysis of all standard cell circuits, the EDFFX1TL register is identified as being the root cause of the complete system failure; in addition, the failure mechanism and fault propagation path are revealed. The results presented in this paper can provide beneficial guidance for a 130 nm processor’s radiation performance evaluation and anti-radiation hardening design.

## Figures and Tables

**Table 1:** Failure thresholds of combinatorial logical cells in the LEON2 processor.

Type	Name	Failure Threshold (krad(Si))
Functional Unit	ADDFXLTL	950
NAND-Type	OAI31XLTL	980
	OAI32XLTL	950
NOR-Type	NOR3XLTL	950
	NOR3BXLTL	950
	NOR4XLTL	850
	NOR4X1TL	950
	AOI211XLTL	960
	AOI221XLTL	960
	AOI222XLTL	960

**Table 2:** Failure thresholds of sequential logical cells in the LEON2 processor.

Name	Failure Threshold (krad(Si))
DFFRX1TL	900
EDFFHQX1TL	1000
MDFFHQX1TL	1000
DFFQX1TL	900
DFFSX1TL	1000
EDFFTRX1TL	800
DFFX1TL	900
EDFFX1TL	770
EDFFXLTL	900
DFFXLTL	900

## Data Availability

The original contributions presented in this study are included in the article. Further inquiries can be directed to the corresponding author(s).

## Abbreviations

The following abbreviations are used in this manuscript:

SMIC	Semiconductor Manufacturing International Corporation
NMOS	N-channel Metal-Oxide Semiconductor
TCAD	Technology Computer-Aided Design
SPICE	Simulation Program with Integrated Circuit Emphasis
CMOS	Complementary Metal-Oxide Semiconductor
SPARC	Scalable Processor Architecture
RISC	Reduced Instruction Set Computer
PMOS	P-channel Metal-Oxide Semiconductor
STI	Shallow Trench Isolation
SRAM	Static Random-Access Memory
RTL	Register Transfer Level
AHB	Advanced High-Performance Bus
